# Transcriptome Analysis of *Aureobasidium pullulans* YQ65 Grown on Yeast Extract Peptone Glucose and Potato Dextrose Agar Media and Quantification of Their Effects on Pullulan Production

**DOI:** 10.3390/foods13223619

**Published:** 2024-11-13

**Authors:** Wan Wang, Jiyun Zhao, Kai Zhang, Zhengran Wang, Jingqiu Ma, Qian Yang, Congyu Lin

**Affiliations:** 1 School of Life Science and Technology, Harbin Institute of Technology, Harbin 150006, China; 17862702273@163.com (W.W.); zjy18845768427@163.com (J.Z.); yangq@hit.edu.cn (Q.Y.); 2 School of Life Science, Ludong University, Yantai 264025, China; zhangkaiown@163.com; 3 Ocean College, Zhejiang University, Zhoushan 316021, China; wangzhengran202106@163.com; 4 School of Food Science and Technology, Jiangnan University, Wuxi 214122, China; majingqiu1224@163.com

**Keywords:** *Aureobasidium pullulans*, pullulan, culture medium, transcriptome analysis, differentially expressed genes

## Abstract

Pullulan is a high-value polysaccharide produced through the fermentation of *Aureobasidium pullulans*. It has significant applications in the fields of food, medicine, environmental science, and packaging. However, the yield, molecular weight, and other characteristics of pullulan can vary depending on the fermentation substrate used. Therefore, it is crucial to analyze the underlying causes of these variations at the molecular level. In this study, we first investigated the morphological differences in *A. pullulans* YQ65 when cultured in YPD and PDA media. The results indicated that different culture media significantly influence the primary cell morphology of *A. pullulans* YQ65, which in turn affects the synthesis of secondary metabolites. Subsequently, we employed different culture media to ferment pullulan and examined the variations in pullulan yield, molecular weight, and biomass. Moreover, FTIR and thermodynamic stability tests were conducted to analyze the differences among pullulans across different culture media. Finally, transcriptome analysis revealed that *A. pullulans* YQ65, when cultured in YPD and PDA media, regulates its growth and metabolism through the expression of key genes that are involved in pathways such as the proteasome, oxidative phosphorylation, metabolism of various secondary metabolites, fatty acid anabolism, carbon metabolism, and amino acid metabolism. The transcriptome results were further validated by assessing the expression of specific genes. This study enhances the understanding of the fermentation differences observed with different substrates in *A. pullulans* and provides valuable insights for optimizing culture substrates. Additionally, it offers guidance for utilizing agricultural and forestry processing waste, as well as food processing by-products, to produce pullulan cost-effectively in the future.

## 1. Introduction

*Aureobasidium pullulans* is an amorphous fungus characterized by a complex cell morphology, which includes yeast-like, spore-like, swollen spore-like, mycelial, and thick-walled spore-like forms [1,2,3]. The morphology of Staphylococcus budding varies under different media and culture conditions [4]. In the past century, research on *Aureobasidium pullulans* primarily focused on classification and identification, the screening of fermentation product strains with practical applications, the optimization of individual strain yields, and the identification of extracellular products, resulting in a substantial body of scientific findings [5,6,7]. With the advent of high-throughput sequencing and gene editing technologies, researchers have begun to investigate the biosynthesis and regulation of extracellular metabolites produced by application-relevant *Aureobasidium pullulans* [8]. Recent studies have highlighted the diverse applications of various *Aureobasidium pullulans*, which not only produce pullulan, melanin, and polymalic acid but also yield a range of extracellular enzymes, including proteases, lipases, and amylases. Nevertheless, pullulan remains the primary product of these strains [9,10].

Pullulan is a water-soluble, amorphous glucan produced through fermentation using cellulose and glucose as primary raw materials, with *Aureobasidium pullulans* serving as the production organism. It is often employed as a model polysaccharide for studies involving water-soluble polysaccharides [11]. The synthesis of pullulan necessitates the presence of carbon and nitrogen sources, while inorganic salts, vitamins, and trace elements also significantly influence the biosynthesis of pullulan [12]. The selection of carbon source is critical, as it directly impacts the yield of the final product. *Aureobasidium pullulans* can utilize various sugars as fermentation carbon sources, including glucose, sucrose, maltose, starch, pectin, cellulose, and lignin [13]. Recent research has focused on identifying suitable carbon sources and enhancing production methods. Additionally, nitrogen sources play a crucial role in regulating the fermentation process of *Aureobasidium pullulans*. Studies indicate that lower concentrations of carbon sources may facilitate greater product accumulation. Common nitrogen sources such as urea, peptone, and ammonium sulfate are typically employed, with ammonium salts and complex nitrogen sources proving to be particularly beneficial for the growth of *Aureobasidium pullulans* and the production of pullulan [14,15,16]. In recent years, there has been considerable interest in reducing the production costs of pullulan. Economically viable agricultural and forestry by-products, such as bagasse, sweet potatoes, and molasses, have been identified as potential feedstocks for the industrial production of pullulan [17,18,19]. However, the yield of pullulan produced using these by-products is low, so the study of culture media is very important.

Different culture media significantly influence the production of compounds that are synthesized by microorganisms, primarily depending on their nutrient composition. The varying ratios of these nutrients also play a crucial role. Consequently, a substantial number of researchers focus on the composition and preparation of culture media, emphasizing the importance of optimizing these conditions [20]. YPD medium, or Yeast Extract Peptone Glucose Medium, is commonly used for culturing yeast in experimental settings. The yeast extract powder supplies essential nutrients, including vitamins, amino acids, and trace elements, that are necessary for yeast growth, while glucose and tryptone provide adequate carbon and nitrogen sources for both yeast growth and compound synthesis. Therefore, YPD medium effectively supports the energy requirements for yeast life processes [21,22]. PDA medium, or Potato Dextrose Agar, is primarily utilized for cultivating fungi. Potatoes are rich in carbohydrates, vitamins, and minerals, offering a diverse array of nutrients for fungal growth. Glucose serves as the primary carbon source, ensuring that PDA medium provides sufficient energy for the life activities of fungi [23].

From the time when *Aureobasidium pullulans* was first discovered to the present, there has been ongoing debate in the scientific community on whether it belongs to fungi or yeast [24]. However, we found during the preliminary experiments that there are many differences between *Aureobasidium pullulan* cultured using YPD and PDA media. The most obvious manifestation is that the yield of pullulan is significantly different, and the biomass is also significantly different. Therefore, it is of great significance to analyze the key differentially expressed genes of *Aureobasidium pullulans* YQ65 in different media from the transcriptome. By understanding the effects of different carbon and nitrogen sources on the synthesis of pullulan, we can further optimize the ingredients in the culture medium. This work establishes a foundation for the efficient production of pullulan using waste resources [25].

## 2. Materials and Methods

### 2.1. Strains and Materials

*Aureobasidium pullulans* YQ65 was isolated and preserved in our laboratory. The culture media utilized in this study included YPD (glucose 20.0 g/L, yeast extract 10.0 g/L, tryptone 20.0 g/L) and PDA (potato extract 200 g/L, glucose 20.0 g/L, pH 6.5). Additionally, 1.5% agar was incorporated into the solid culture medium.

### 2.2. Cultivation and Morphological Observation of Aureobasidium pullulans YQ65

A ring of *Aureobasidium pullulans* YQ65 was selected from the glycerol storage tube and spread onto a solid plate for activation. The plate was incubated at 28 °C for 48 h, after which a single colony was selected and transferred to a liquid culture medium. This culture was maintained at 28 °C with shaking at 180 rpm for 24 h to serve as the primary seed culture medium. Subsequently, the primary culture was transferred at a 3% inoculation rate to a secondary seed culture medium, which was then incubated at 28 °C with shaking at 180 rpm for an additional 24 h. The secondary seed culture of *Aureobasidium pullulans* YQ65 was subsequently transferred to a liquid culture medium and incubated at 28 °C with shaking at 220 rpm for 8 d. An appropriate volume of fungus culture was sampled daily, and the cell morphology of *Aureobasidium pullulans* YQ65 was observed using an optical microscope (XSP-14C, Shanghai Batuo Instrument Co., Ltd., Shanghai, China) [25].

### 2.3. Determination of Pullulan Yield, Molecular Weight, and Biomass

*Aureobasidium pullulans* YQ65 was fermented in PDA and YPD media for 8 d. The biomass, pullulan yield, and molecular weight were subsequently assessed. The fermentation broth was centrifuged at 5000 rpm for 5 min (high-speed benchtop centrifuge, Hunan Kaida KH23A, Kaida, Changsha, China), and the resulting precipitate was collected and dried at 60 °C for 24 h. Biomass was quantified by weighing, and two volumes of ethanol were added to the separated supernatant, which was then incubated at 4 °C overnight for alcohol precipitation. The precipitate was collected via centrifugation at 5000 rpm and dried at 60 °C for 24 h. The pullulan yield was determined by weighing 0.02 g of pullulan, which was dissolved in 4 mL of 0.1 M NaNO_3_ solution for 10 min, followed by filtration through a 0.45 μm filter membrane. The molecular weight of the pullulan was analyzed using Agilent 1260 gel permeation chromatography (Agilent, Santa Clara, CA, USA) (flow rate: 1 mL/min; detector G1362A) [26,27,28].

### 2.4. Differential Analysis of Pullulan by FTIR

After culturing *Aureobasidium pullulans* YQ65 in PDA and YPD media for 6 d, the culture was subjected to centrifugation at 5000 rpm to collect the liquid. Subsequently, an equal volume of ethanol was added to the liquid, and the mixture was allowed to precipitate at 4 °C overnight. The resulting precipitate was collected through centrifugation at 5000 rpm and dried at 60 °C for 24 h. A precise amount of 5.0 mg of pullulan, produced using PDA and YPD media, was weighed and uniformly ground with 50.0 mg of dry KBr powder in a mortar. The resulting mixture was then compressed into tablets and analyzed using FTIR (Spectrum 100, PerkinElmer, Shelton, CT, USA) with a scanning range of 4000 to 450 cm^−1^ and a resolution of 4 cm^−1^ [25].

### 2.5. Analysis of Differences in Thermodynamic Stability

Thermogravimetric analysis (TGA) was conducted using a Mettler-Toledo TGA-SDTA851e derivatizer. A sample weighing 5.0 mg was heated within a temperature range of 30–800 °C, with nitrogen flowing at a rate of 20 mL/min and a heating rate of 10 °C/min. Differential scanning calorimetry (DSC) analysis was performed with a Mettler-Toledo DSC (Mettler-Toledo, Zurich, Switzerland) and STARe software (version 9.1). The 5.0 mg sample was encapsulated in an aluminum pan featuring a perforated lid to facilitate the escape of volatiles. Nitrogen was also introduced at a rate of 20 mL/min and a heating rate of 10 °C/min within the temperature range of 30–220 °C [29,30]. The operating parameters were maintained consistently across all samples to ensure the comparability of the data, and recordings were repeated under the same heating rate to verify reproducibility.

### 2.6. Transcriptome Sequencing and Analysis

Total RNA was extracted from *Aureobasidium pullulans* cultured in YPD and PDA media using Trizol (Thermo Fisher, Waltham, MA, USA), and its quality was assessed. Subsequently, ribosomal RNA (rRNA) was removed, followed by the synthesis of the first and second strands of complementary DNA (cDNA). The ends of the cDNA were repaired, adapters were ligated, and fragments were screened following USER enzyme digestion. PCR amplification was then conducted, and the resulting products were purified and sequenced on the Illumina platform after quality inspection. After acquiring the raw data, FASTP was employed to filter out low-quality reads, defined as those where the proportion of bases with a quality value of Q ≤ 20 exceeded 50%. The filtered clean reads were then aligned with the reference genome for bioinformatics analyses, including differential gene expression, functional annotation of differentially expressed genes, and functional enrichment analysis [31,32].

### 2.7. Primer Design and Gene Expression Verification

The cells cultured in YPD and PDA media were centrifuged, precipitated, and rinsed twice with DEPC-treated water before being ground into a powder using liquid nitrogen. Total RNA was extracted from the cells using Trizol reagent. To maintain RNA stability, the extraction process was conducted at low temperatures, and reverse transcription experiments were performed promptly to synthesize complementary DNA (cDNA). To validate the accuracy of the transcriptome sequencing results, representative differentially expressed genes were selected for quantitative fluorescence PCR. Primers were designed to quantitatively analyze the expression of hexokinase (Gluk), UDP-glucose phosphorylase (Ugp), glucosyltransferase (Ugt), pullulan synthase (Pul), glutathione S-transferase (GST), and peroxidase (POD). The primer sequences are listed in Supplementary Table S1 [33].

### 2.8. Statistical Analysis

All data were statistically analyzed using SPSS version 19.0 (IBM Corporation, Armonk, NY, USA). Duncan’s multiple range test was employed to compare the means of the data. A *p*-value of less than 0.05 was considered statistically significant.

## 3. Results and Discussion

### 3.1. Comparison of the Morphology of Aureobasidium Pullulans YQ65 Cultured on Different Media

To investigate the morphological characteristics of *Aureobasidium pullulans* YQ65 during its growth in YPD and PDA media, we cultured the organism for 8 d and observed its morphology throughout the culture period using an optical microscope (×40).

**Figure 1:**
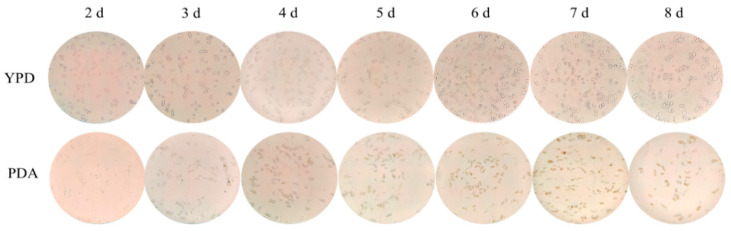
Optical microscope images of *Aureobasidium pullulans* YQ65 in different culture media (×40).

As illustrated in Figure 1, under a 40× optical microscope, *Aureobasidium pullulans* YQ65 cultivated in YPD medium predominantly exhibits oval yeast-like cells, with a minority of thick-walled spores being present and no observable mycelium. In PDA medium, both yeast-like cells and thick-walled spores coexist during the early stages of fermentation. As the fermentation progresses, there is a significant increase in the number of thick-walled spores, mycelium becomes apparent, and pigment granules are observed within the cells. In the later stages of fermentation in PDA medium, pigment granules are released into the fermentation liquid following cell lysis. The observed changes in cell morphology across various culture media suggest that the morphology of *Aureobasidium pullulans* YQ65 can be modulated by the choice of culture medium. YPD was identified as the optimal medium for promoting the growth and proliferation of yeast-like cells of *Aureobasidium pullulans* YQ65. Conversely, PDA medium is most effective for the development of thick-walled spore cells of this strain, which enhances the synthesis of various secondary metabolites [25]. Previous studies have primarily adopted a macroscopic perspective; however, this study delves deeper into the underlying mechanisms, demonstrating that various culture media significantly influence the primary cell morphology of *Aureobasidium pullulans* YQ65, which in turn affects the synthesis of secondary metabolites.

### 3.2. Differences in Yield, Molecular Weight, and Biomass of Pullulan Produced by Fermentation in Different Culture Media

**Figure 2:**
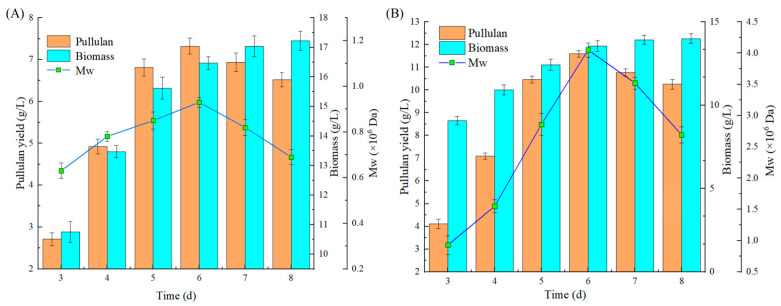
The yield, molecular weight, and biomass of pullulan produced by fermentation in different media: (**A**) The yield, molecular weight, and biomass of pullulan produced by fermentation in YPD medium; (**B**) the yield, molecular weight, and biomass of pullulan produced by fermentation in PDA medium.

Different fermentation substrates significantly influence microbial fermentation processes. These influences not only affect cell morphology but also have a substantial impact on the levels of secondary metabolites [8]. As illustrated in Figure 2, there are notable differences in the yield, molecular weight, and biomass of pullulan produced by the fermentation of *Aureobasidium pullulan* YQ65 in two distinct media. Figure 2A,B demonstrate that in both culture media, the yield and molecular weight of pullulan initially increase and then decrease, reaching their maximum values on the sixth day. This fluctuation may be attributed to the hydrolysis of pullulan by enzymes such as α-amylase, glucoamylase, and isopululanase, which leads to a reduction in yield, while the biomass continues to rise. Over time, the biomass increases at a gradually slowing rate, primarily due to the depletion of nutrients in the substrate. In comparison, the yield of pullulan produced in PDA medium (11.59 g/L) was significantly higher than that produced in YPD medium (7.32 g/L). This pattern is also observed in the molecular weight; the molecular weight of pullulan produced in PDA medium (4.07 × 10^6^ Da) is significantly greater than that of pullulan produced in YPD medium (0.92 × 10^6^ Da). Interestingly, the biomass exhibits an inverse relationship to the yield and molecular weight; the biomass produced in YPD medium (17.22 g/L) is significantly higher than that produced in PDA medium (13.99 g/L). This phenomenon indicates that YPD medium is more conducive to the growth of *Aureobasidium pullulans* YQ65. In the fermentation system, microbial cells proliferate continuously, consuming carbon and nitrogen sources in the substrate, which affects metabolite production. Conversely, PDA medium is more favorable for the synthesis of secondary metabolites, resulting in a significantly higher yield of pullulan compared to YPD medium.

### 3.3. Analysis of the Difference in Pullulan Production in Different Culture Media by FTIR

**Figure 3:**
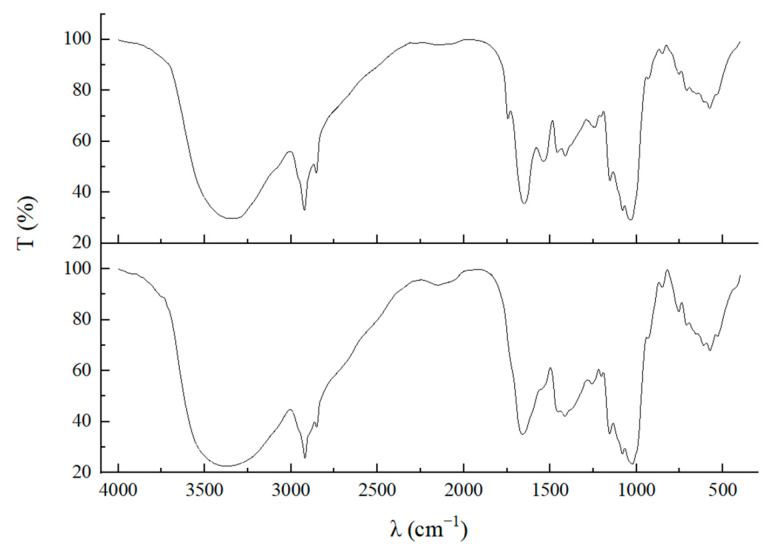
FTIR of pullulan produced by fermentation in different media.

An FTIR spectrometer is an analytical instrument that is capable of determining the molecular structure of a substance and identifying compounds based on vibrational and rotational information of the molecules. This instrument effectively analyzes the vibrations of chemical bonds associated with fermented polysaccharides, enabling the identification of the sample’s composition and differences through characteristic peaks [25]. As illustrated in Figure 3, the characteristic peaks of polysaccharide molecules produced using YPD and PDA media include the V_C=O_ peak at 1022 cm^−1^ and the D-pyranose ring vibration peak at 933 cm^−1^. Notably, between 700 cm^−1^ and 1000 cm^−1^, distinct sugar characteristic absorption peaks can be observed. The absorption bands at 2927, 1724, 1161, and 933 cm^−1^ correspond to C-H, O-C-O, and C-O stretching vibrations, respectively. In this polypyran polymer, oxygen atoms adjacent to the C5–C6 bond can form primary C-O-H, resulting in a band within the range of 1200–1400 cm^−1^. The characteristic peaks of these two polysaccharide samples closely resemble the pullulan characteristic peaks documented in the literature, confirming that the polysaccharides produced using YPD and PDA media are both pullulan and exhibit no significant differences in characteristics. This indicates that variations in fermentation substrates do not alter the chemical bonds of the product, thereby preserving the structural integrity [3].

### 3.4. Analysis of the Difference in Thermodynamic Stability of Pullulan Produced in Different Culture Media

**Figure 4:**
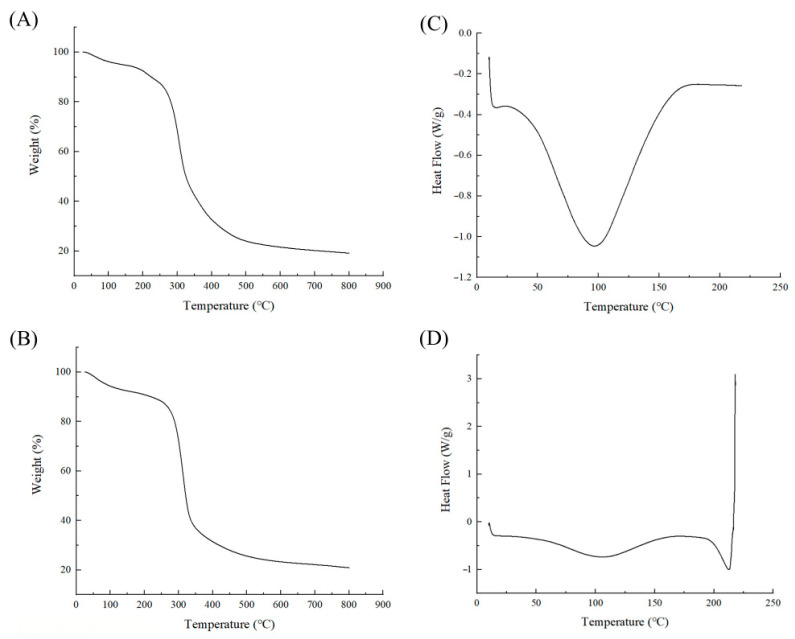
Thermodynamic stability analysis. (**A**) TGA curve of pullulan produced in YPD medium; (**B**) TGA curve of pullulan produced in PDA medium; (**C**) DSC curve of pullulan produced in YPD medium; (**D**) DSC curve of pullulan produced in PDA medium.

It is essential to investigate the differences in pullulan fermentation production across various culture media concerning thermodynamic stability. Such an examination can elucidate differences in molecular structure and chemical properties, providing valuable insights for application development. Figure 4A,B present the thermogravimetric analysis (TGA) curves of different pullulan polysaccharides, illustrating the weight loss of pullulan produced through fermentation in YPD and PDA media during the heating process. As the temperature increases, the mass loss rate of both samples progressively rises. Notably, at approximately 280 °C, a turning point occurs, where the minor weight loss below this temperature can be attributed to water evaporation and the degradation of impurities. Above 280 °C, both samples exhibit rapid weight loss, which can be attributed to the decomposition of the pullulan. The shapes of curves A and B are similar, with individual variances likely stemming from impurities in the samples. There were no significant differences observed in the pyrolysis process of pullulan produced in YPD and PDA media. Figure 4C,D display the differential scanning calorimetry (DSC) curves, which illustrate the thermal changes in pullulan produced by fermentation in YPD and PDA media during the heating process. Pullulan from YPD fermentation exhibits a melting point around 100 °C, indicated by a prominent endothermic peak. In contrast, the melting peak of pullulan produced in PDA medium occurs at approximately 110 °C and is less pronounced, suggesting that this sample does not melt as readily at this temperature. A relatively sharp endothermic peak associated with thermal decomposition appears near 210 °C, with no observable thermal effect occurring between 0 and 220 °C, as shown in Figure 4C. The results indicate that the thermodynamic stability of pullulan produced in PDA medium is significantly superior to that of pullulan produced in YPD medium, potentially due to differences in the degree of polymerization among the molecules. This is further substantiated by the molecular weight data presented in Section 3.2.

### 3.5. Transcriptome Sequencing Gene Statistics and Sample Relationships

**Figure 5:**
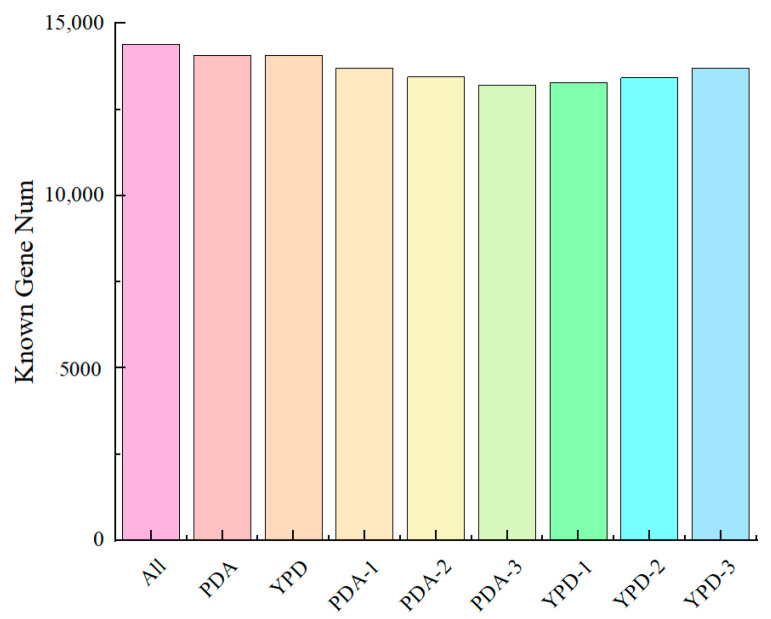
Gene identification statistics.

As illustrated in Figure 5, the reference genome comprises a total of 14,397 genes, whereas this sequencing identified 14,099 genes, representing 97.93% of the total. Specifically, the PDA group measured 14,062 genes, accounting for 97.67%, while the YPD group identified 14,079 genes, corresponding to 97.79%. The detection counts for each sample are detailed in Supplementary Table S1. These findings confirm the appropriateness of the reference genome selection and its suitability for subsequent analyses of differentially expressed genes.

**Figure 6:**
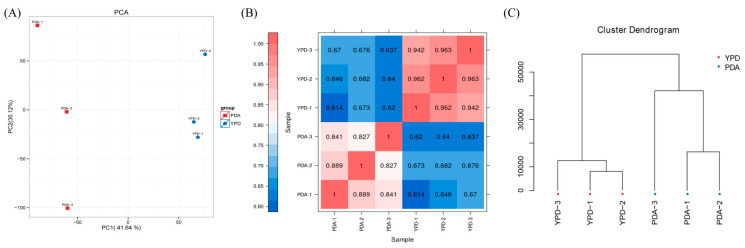
Sample relationships. (**A**) Principal component analysis; (**B**) correlation heat map; (**C**) sample clustering map.

Based on the expression profiles of known genes in each sample, we conducted analyses utilizing principal component analysis (PCA), Pearson correlation coefficients, and additional methods to comprehensively evaluate the repeatability among samples, thereby facilitating the exclusion of outlier samples [32]. We utilized R programming to extract the numerical values of each sample along the PC1 and PC2 principal components, subsequently generating a two-dimensional coordinate plot, as illustrated in Figure 6A. The results indicate minimal differences within each sample group, with no outlier samples identified. PCA effectively reflects the relationships among samples, while the correlation coefficient quantitatively measures the strength of these relationships. We calculated the expression levels of all genes between each pair of samples and computed the Pearson correlation coefficients accordingly. As depicted in Figure 6B, samples within the same group exhibit darker colors, signifying stronger correlations, whereas samples from different groups display lighter colors, indicating weaker correlations. This analysis facilitates the identification of differentially expressed genes. Notably, Figure 6C presents a sample clustering diagram, which reveals that the YPD and PDA groups occupy distinct branches, highlighting significant differences between the groups. This finding underscores the meaningfulness of the experiment and confirms that the correlation among samples within the same group is stronger, thereby corroborating the experimental results regarding pullulan yield, molecular weight, and biomass.

### 3.6. Gene Expression Analysis

**Figure 7:**
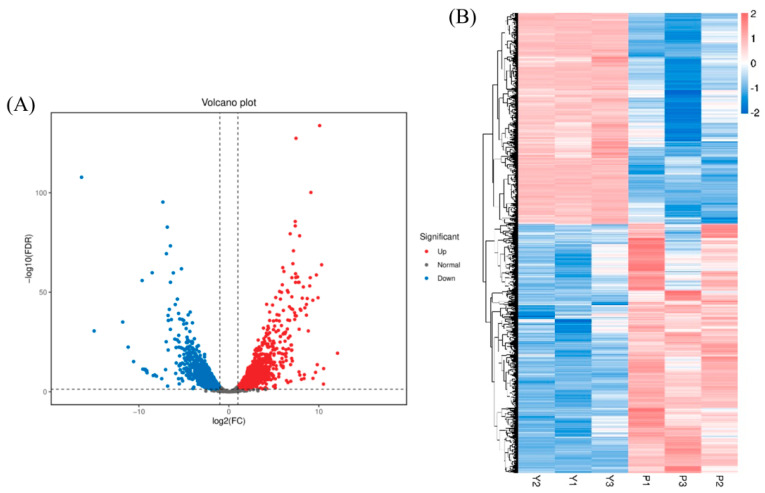
Gene expression analysis. (**A**) Volcano plot of differences between groups; (**B**) heat map of all differential gene expression levels.

Figure 7A presents a volcano plot illustrating the comparative transcriptome gene expression between YPD and PDA media. In this analysis, a total of 4144 differentially expressed genes were identified, with 2240 genes exhibiting upregulation and 1904 genes demonstrating downregulation. These differentially expressed genes displayed distinct transcriptional patterns across the two media. Figure 7B provides a visual representation of the hierarchical clustering of transcript levels for the differentially expressed genes. Notably, among all the up- and downregulated genes, 60.5% exhibited fold changes between 2 and 4, 22.6% between 4 and 8, 7.9% between 8 and 16, and 8.9% showed changes greater than 16-fold at the mRNA level. Many of these differentially expressed genes are associated with fungal metabolic functions, including multicopper oxidase (upregulated 776-fold), cytochrome P450 monooxygenase (upregulated 124-fold), tetrahydroxynaphthalene reductase (upregulated 48-fold), and polyketide synthase (upregulated 22-fold). Notably, polyketide synthase and tetrahydroxynaphthalene reductase are essential enzymes in the melanin synthesis pathway, indicating that the observed color differences in the PDA fermentation broth are significantly correlated with the expression levels of these enzymes.

### 3.7. Functional Annotation of Differentially Expressed Genes

The coding sequences (CDSs) derived from transcriptome sequencing were analyzed using the GO and KEGG databases. The functional information of the differentially expressed genes was then summarized and evaluated based on the annotations obtained from these databases.

#### 3.7.1. GO Analysis

**Figure 8:**
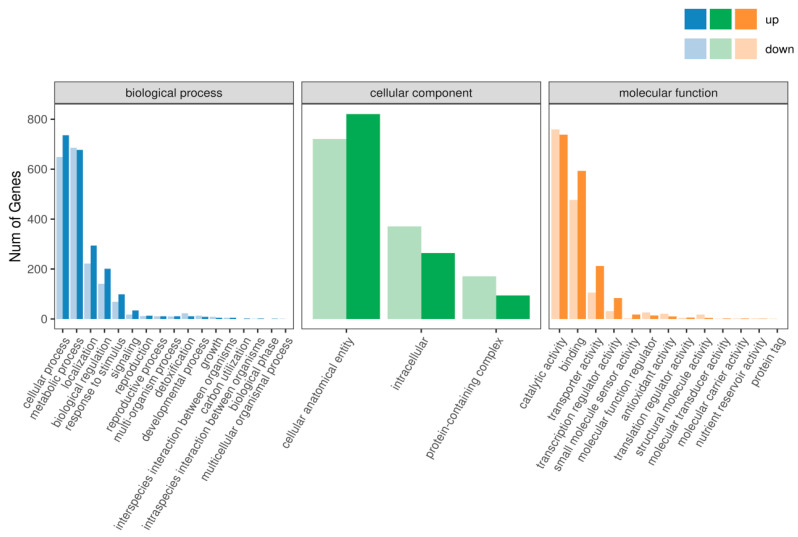
Enrichment of biological process, cellular component, and molecular function in differentially expressed genes.

Through pathway analysis of differentially expressed genes, significant changes under comparative conditions can be observed. As shown in Figure 8, an enrichment analysis of the GO functions related to differentially expressed genes reveals the following patterns: in the comparison between the YPD and PDA groups, the differentially expressed genes in biological processes primarily relate to cellular processes, metabolic processes, localization, biological regulation, and response to stimuli. When comparing the YPD group with the PDA group, we observed that the upregulated genes associated with cellular processes, localization, biological regulation, and response to stimuli were 736, 294, 201, and 99, respectively, significantly exceeding the downregulated genes, which were 649, 222, 141, and 69. In contrast, the number of upregulated genes related to metabolic processes was 677, which was lower than the 686 downregulated genes. Additionally, genes associated with signal transduction, reproduction, production processes, multi-organism processes, detoxification, developmental processes, and growth also exhibited responses to the YPD and PDA groups.

The differences in molecular functions among genes primarily pertain to catalytic activity, binding, transport activity, transcriptional regulator activity, and small molecule sensor activity. A comparison between the YPD and PDA groups reveals that the number of upregulated genes for binding, transport activity, transcriptional regulator activity, and small molecule sensor activity (593, 212, 84, and 18, respectively) significantly exceeds that of downregulated genes (477, 106, 31, and 3). In contrast, the number of upregulated genes in catalytic activity (738) is lower than that of downregulated genes (759). Additionally, the number of differentially expressed genes related to oxidoreductase activity and transmembrane transporter activity exceeds 150. Furthermore, genes associated with antioxidant activity, molecular function regulators, and structural molecule activity also respond to the differences between the YPD and PDA groups.

#### 3.7.2. KEGG Analysis

**Figure 9:**
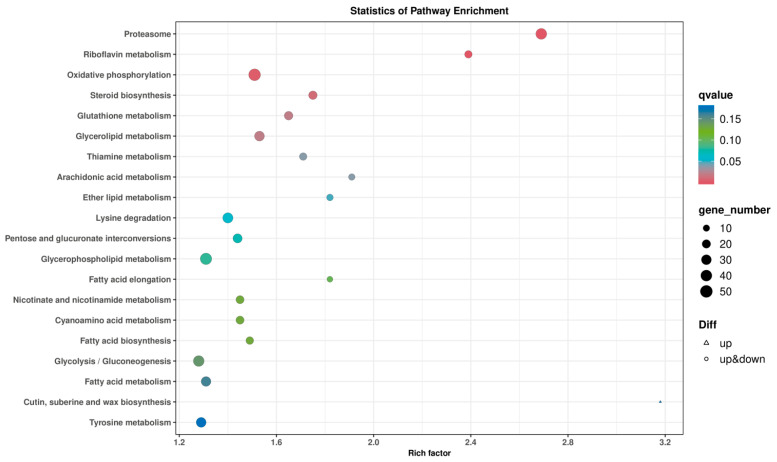
KEGG analysis of differentially expressed genes.

The growth, development, and metabolism of microorganisms rely on the coordination of complex metabolic pathways. Conducting pathway enrichment analysis on differentially expressed genes can provide deeper insights into the functions of these metabolic pathways and their interrelationships. As shown in Figure 9, the top 20 enriched pathways in the comparison between YPD and PDA include the proteasome, oxidative phosphorylation, lysine degradation, glycosylphosphatidylinositol metabolism, and glycolysis/gluconeogenesis, each involving over 40 differentially expressed genes. Additionally, pathways such as riboflavin metabolism, steroid biosynthesis, glycerolipid metabolism, glutathione metabolism, thiamine metabolism, fatty acid biosynthesis, cyanate metabolism, nicotinic acid and nicotinamide metabolism, fatty acid metabolism, and tyrosine metabolism also include more than 20 differentially expressed genes. Through this analysis, we found that pathways with a significant number of differentially expressed genes, such as the proteasome, oxidative phosphorylation, various secondary metabolite metabolisms, fatty acid synthesis metabolism, carbon metabolism, and amino acid metabolism, are notably affected in both culture media. The metabolites produced from these pathways also influence the growth and development of microorganisms and play a crucial role in the synthesis of pullulan.

### 3.8. Validation of Key Genes

Significant differences exist in the cell morphology of *Aureobasidium pullulans* when cultured in YPD medium compared to PDA medium. In YPD medium, the strain predominantly exhibits yeast-like cells, while in PDA media, chlamydospore cells are more prevalent. These two morphologies are characteristic of *Aureobasidium pullulans*, which is the primary producer of the pullulan, significantly influencing its yield [25].

During the biosynthesis of pullulan, several key enzymes are involved, including hexokinase (Gluk), uridine diphosphate glucose pyrophosphorylase (Ugp), glucosyltransferase (Ugt), and pullulan synthase (Pul). Hexokinase and related enzymes convert carbon sources into glucose-6-phosphate [17]. Subsequently, α-phosphoglucomutase catalyzes the conversion of glucose-6-phosphate to glucose-1-phosphate. UDP-glucose is synthesized from glucose-1-phosphate and UDP through the action of UDPG-pyrophosphorylase, which also produces pyrophosphate. Glucosyltransferase is responsible for generating panose (Glc-α-1→6-Glc-α-1→4Glc) and isomaltose (Glc-α-1→6-Glc), both of which are crucial for the synthesis of pullulan. Pullulan synthase facilitates the linkage of isomaltose to form pullulan. Moreover, *Aureobasidium pullulans* is a highly resilient yeast-like fungus that utilizes cell differentiation as a strategy to withstand environmental stress. The observed differences in the differentiation of chlamydospore and yeast-like cells across the two media may be attributed to oxidative stress [6]. Consequently, glutathione S-transferase (GST) and peroxidase (POD) were selected for quantitative analysis. GST catalyzes the reaction between glutathione (GSH) and reactive oxygen species (ROS), thereby reducing the ROS concentration and protecting cells from oxidative damage. GSTs also play a role in regulating the expression of other antioxidant enzymes, such as superoxide dismutase (SOD) and peroxidase (POD), enhancing the overall resistance of fungal cells to oxidative stress. POD protects cells from oxidative damage by scavenging hydrogen peroxide, bolstering antioxidant defenses, regulating signaling pathways, and promoting cell survival.

**Figure 10:**
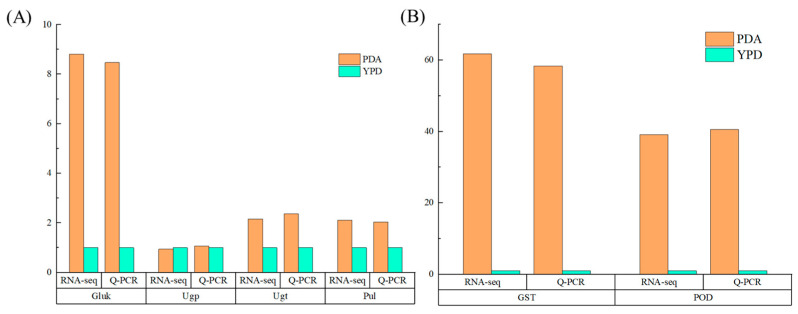
Verification of key genes. (**A**) Genes related to pullulan synthesis; (**B**) genes related to oxidative stress.

The results of the fluorescence quantitative PCR for differential gene expression are presented in Figure 10. The expression trends of the selected differential genes align with those obtained from transcriptome sequencing, confirming the reliability of the transcriptome sequencing results.

With the rapid growth of the global population, the demand for consumer goods continues to rise. Since it is a high-quality food ingredient, research on pullulan has garnered significant attention from the scientific community, making efficient production a top priority. Currently, numerous scholars are enhancing the synthesis efficiency of pullulan by screening high-yield strains and employing mutagenesis techniques [6]. Additionally, some experts are increasing pullulan production by optimizing the fermentation processes and equipment. Consequently, the culture medium, as a synthetic substrate, warrants considerable attention. Early experiments indicated notable differences in the pullulan produced through fermentation in YPD medium compared to PDA medium [16]. In this study, we cultured *Aureobasidium pullulans* YQ65 in both YPD and PDA media. As illustrated in Figure 1, significant morphological differences in the strains were observed over a period of 2 to 8 days. Most strains cultured in YPD medium displayed an elliptical morphology, characteristic of yeast, with few chlamydospores and an absence of mycelium. In contrast, both forms of the strains coexisted in PDA medium, where mycelium production developed over time. These findings indicate that varying culture media significantly affect the strain morphology, resulting in a series of consequential effects. Figure 2 highlights the differences in yield, molecular weight, and biomass of pullulan produced in various media. This observation concretely reflects the differences shown in Figure 1, underscoring that diverse media can markedly affect microbial growth and metabolism [25]. For pullulan synthesized in different culture media, the results obtained through FTIR and thermodynamic stability experiments are presented in Figure 3 and Figure 4. The findings confirm that all samples produced pullulan; however, the pullulan derived from PDA medium exhibited superior thermal stability compared to that produced in YPD medium. This suggests that the pullulan from the two media possesses distinct molecular structures and chemical properties, potentially attributable to variations in molecular weight. A higher molecular weight correlates with an increased degree of polymerization among monomers, reinforcing the differences in molecular weight depicted in Figure 2 [26]. The discrepancy in thermal stability offers valuable insights for the application of pullulan across different fields. For instance, in the food industry, enhanced thermal stability is advantageous for high-temperature processing, whereas, in environmental protection, excessively high thermal stability may hinder the occurrence of secondary degradation.

To elucidate the genetic basis underlying variations in pullulan yield, molecular weight, and biomass production across different culture media used in prior experiments, this study performed transcriptome sequencing on *Aureobasidium pullulans* YQ65 cultured in various media. The analysis revealed that the differentially expressed genes associated with biological processes were primarily related to cellular processes, metabolic processes, localization, biological regulation, and responses to stimuli. Furthermore, the differentially expressed genes linked to molecular functions predominantly pertained to catalytic activity, binding, transport activity, transcriptional regulation, and small molecule sensing. These findings suggest that alterations in the expression levels of these genes significantly influence the growth and metabolism of *Aureobasidium pullulans* YQ65, with key components in the culture medium playing a critical role [28]. The KEGG analysis indicated the enrichment of multiple pathways characterized by a substantial number of differentially expressed genes, including those that are involved in proteasome function, oxidative phosphorylation, the metabolism of various secondary metabolites, fatty acid anabolism, carbon metabolism, and amino acid metabolism. This underscores that different culture media modulate metabolic regulation through gene expression. These genes have been validated in the key pathways of pullulan production. Consequently, it is evident that varying culture media significantly impact pullulan synthesis [33]. Future research may focus on optimizing pullulan production by adjusting fermentation substrates, balancing carbon and nitrogen source ratios, and modifying inorganic salt concentrations. Additionally, this approach could provide insights into waste reduction and cost minimization by utilizing agricultural and forestry processing by-products as cost-effective substrates for pullulan production.

## 4. Conclusions

This study initially investigated the growth conditions and morphological characteristics of *A. pullulan* YQ65 across various media. It compared the yield, molecular weight, and biomass of pullulan produced through fermentation in YPD medium with those in PDA medium. Additionally, the study analyzed the similarities and differences between the two media using FTIR and thermodynamic stability assessments. The results demonstrated that PDA medium was more favorable for pullulan production, while YPD medium was more effective for cultivating *A. pullulans*. Subsequently, a transcriptome sequencing analysis was conducted on *Aureobasidium pullulan* YQ65, which was fermented to produce pullulan in both YPD and PDA media. The findings revealed that several pathways, including oxidative phosphorylation, fatty acid anabolism, carbon metabolism, and amino acid metabolism, exhibited significant differences in differentially expressed genes, leading to the observed variations. The quantitative analysis of key genes further supported these findings. This study offers significant insights for the future application of genetic engineering in the development of engineered strains aimed at enhancing pullulan production. Additionally, it serves as a valuable reference for optimizing substrates used in the production of pullulan from resource waste.

## Data Availability

The original contributions presented in the study are included in the article/Supplementary Material, further inquiries can be directed to the corresponding author.

## Supplementary Materials

The following supporting information can be downloaded at https://www.mdpi.com/article/10.3390/foods13223619/s1: Table S1: Primers used in this study.
