# Supplementary material for: Transcriptome Analysis of Aureobasidium pullulans YQ65 Grown on Yeast Extract Peptone Glucose and Potato Dextrose Agar Media and Quantification of Their Effects on Pullulan Production
Source: Foods. 2024 Nov 13;13(22):3619. doi: 10.3390/foods13223619 (PMC11593368; doi:10.3390/foods13223619)
Supplement: Supplementary file 1 [file foods-13-03619-s001.zip › foods-3300010-supplementary.pdf]

foods

Supplementary

Table S1. Primers used in this study.

Gene ID	Name	Sequence (5′ to 3′)
Gluk	GlukF	CTTCAGCGACGACAACAGCAG
	GlukR	CGTGAACGCCGTAGTCCTTGT
Ugp	UgpF	ACGAGGGCTGTGAGTCC
	UgpR	GCCCTCGTAGTCAATGATGGT
Ugt	UgtF	GACCAGCACAAGCGGGT
	UgtR	GCGCGGGTACGTTGGC
Pul	Pul1F	CTACCCCTATCGTCAAAC
	Pul1R	TGGGTTGGTAGTAGGGAT
GST	GSTF	CTTGCTTGAAGAACTCGCCCTC
	GSTR	CGCTCGAGCGGATCTTGG
POD	PODF	CTGGTGGTGCCGATGGATC
	PODR	GTGCTGGTATCCTTGCGAC
